# Sensor Data Required for Automatic Recognition of Athletic Tasks Using Deep Neural Networks

**DOI:** 10.3389/fbioe.2019.00473

**Published:** 2020-01-21

**Authors:** Allison L. Clouthier, Gwyneth B. Ross, Ryan B. Graham

**Affiliations:** School of Human Kinetics, Faculty of Health Sciences, University of Ottawa, Ottawa, ON, Canada

**Keywords:** human activity recognition, wearable sensors, machine learning, neural network, movement screens

## Abstract

Movement screens are used to assess the overall movement quality of an athlete. However, these rely on visual observation of a series of movements and subjective scoring. Data-driven methods to provide objective scoring of these movements are being developed. These currently use optical motion capture and require manual pre-processing of data to identify the start and end points of each movement. Therefore, we aimed to use deep learning techniques to automatically identify movements typically found in movement screens and assess the feasibility of performing the classification based on wearable sensor data. Optical motion capture data were collected on 417 athletes performing 13 athletic movements. We trained an existing deep neural network architecture that combines convolutional and recurrent layers on a subset of 278 athletes. A validation subset of 69 athletes was used to tune the hyperparameters and the final network was tested on the remaining 70 athletes. Simulated inertial measurement data were generated based on the optical motion capture data and the network was trained on this data for different combinations of body segments. Classification accuracy was similar for networks trained using the optical and full-body simulated inertial measurement unit data at 90.1 and 90.2%, respectively. A good classification accuracy of 85.9% was obtained using as few as three simulated sensors placed on the torso and shanks. However, using three simulated sensors on the torso and upper arms or fewer than three sensors resulted in poor accuracy. These results for simulated sensor data indicate the feasibility of classifying athletic movements using a small number of wearable sensors. This could facilitate objective data-driven methods that automatically score overall movement quality using wearable sensors to be easily implemented in the field.

## Introduction

Movement screens are used to assess the overall movement quality of an athlete. Typically, the athlete will perform a series of movements while a trained rater visually observes and scores the movements. The goals of movement screens are to predict injury risk and identify performance deficits that can be targeted in training. While interrater and intrarater reliabilities for movement screens such as the Functional Movement Screen (FMS™) are good (Minick et al., 2010; Teyhen et al., 2012), interrater reliability for subtest components can be poor and dependent on rater experience (Smith et al., 2013; Gulgin and Hoogenboom, 2014; Bonazza et al., 2017). Furthermore, concerns have been raised that grading criteria can be somewhat ambiguous (Frost et al., 2015; Bonazza et al., 2017) and scores may not be sensitive enough to detect movement abnormalities (Clifton et al., 2013). Recent work has aimed to develop objective scoring methods for movement screens (Ross et al., 2018). Data-driven approaches have the potential to improve the repeatability of scoring and increase the ability to detect subtle differences in movement patterns. However, current methods require manual processing of motion capture data before scoring can be performed, including cropping trials to isolate each movement. Additionally, the reliance on optical motion capture could be a barrier to implementation of these methods in the field.

Wearable sensors are an attractive alternative to optical motion capture for motion analysis applications. They are cost-effective and portable, allowing for the collection of motion data outside of a laboratory and over large capture volumes. Furthermore, wearable sensors have the potential to be less cumbersome than optical markers depending on the number and placement of sensors. Previous work investigated optimal placement and number of sensors to classify activities of daily living (Pannurat et al., 2017), everyday activities (Kern et al., 2003; Olguin and Pentland, 2006; Atallah et al., 2011; Cleland et al., 2013), and fall detection (Gjoreski et al., 2011). However, which sensors are necessary to best classify movement screening tasks remains unclear.

Human activity recognition is an area of research that seeks to automatically identify human activities by applying machine learning techniques to motion data. Methods have been developed to classify movements including hand gestures (Kim and Toomajian, 2016), activities of daily living (Hammerla et al., 2016), and movements typical in various sports (Nguyen et al., 2015; Kautz et al., 2017). Previously, activity recognition methods employed techniques that required hand-selected features as input (Bulling et al., 2014). However, convolutional neural networks (CNNs), a type of deep neural network (DNN), are now commonly used to automatically generate features through deep learning (Zeng et al., 2014; Yang et al., 2015; Lee et al., 2017). CNNs have shown promising results in activity recognition; however, they are unable to capture time dependencies. Recurrent neural networks are a type of neural network that include a memory component that allows them to model temporal dependencies. The combination of CNNs to extract features with long-short-term memory (LSTM) recurrent networks to capture temporal dependences has provided improved classification performance over CNNs alone (Ordóñez and Roggen, 2016).

The use of deep neural networks in movement screens would allow for a continuous data collection during a movement screen. Individual movements could then be automatically identified and segmented as a preparation for further analysis or scoring. This would decrease the manual effort required for the analysis process and increase the utility of these objective measurement techniques. The ability to perform the movement classification and scoring based on data from a minimal set of wearable sensors would further increase the applicability of data-driven movement screens. Therefore, our first aim was to use a deep neural network to identify when movements typical of movement screens occur within motion data. Our second aim was to compare networks trained using optical motion capture data with those trained using data available from wearable sensors.

## Methods

### Data Collection and Processing

Optical motion capture data were collected from 417 athletes performing a series of movement tests by Motus Global (Rockville Center, NY). The athletes competed in a variety of sports, including baseball, basketball, soccer, golf, tennis, track and field, squash, cricket, lacrosse, football, and volleyball. They ranged in skill level from recreational athletes to those playing in major professional sports leagues (e.g., NBA, MLB, PGA, etc.). Participants provided informed consent for future use of their data for research before completing the protocol. The secondary use of the data was approved by the University of Ottawa Research Ethics Board. Forty-five retroreflective markers were placed on the athlete for motion tracking (Ross et al., 2018) and data were recorded at 120 Hz using an eight-camera Raptor-E (Motion Analysis, Santa Rosa, CA) motion capture system. Each athlete performed a series of movement tests consisting of 21 unique movements. The 13 movements most likely to challenge mobility and stability were selected for analysis in this study, including hop down right/left (HDR, HDL), bird-dog right/left (BDR, BDL), drop jump (DJ), T-balance right/left (TBR, TBL), step-down right/left (SDR, SDL), L-hop right/left (LHR, LHL), and lunge right/left (LR, LL) (Ross et al., 2018). Individual trials were collected for each movement.

Start and end time points were manually identified for each trial (Ross et al., 2018) for use as a ground truth of when each activity was performed. The optical motion data used in the analysis (OPT) included global x, y, z coordinates for 45 markers. To simulate data that can be obtained using inertial measurement units (sIMU), marker trajectories were processed in Visual3D (C-Motion, Inc., Germantown, MD) and global angular orientation Euler angles and the Euclidean norm of the center of mass linear acceleration and angular velocity for each body segment were calculated. The Euclidean norm of the velocity and acceleration was used to reduce the reliance on accurate sensor alignment. Accelerations and velocities were low-pass filtered at 15 Hz with a zero-lag second order Butterworth filter.

### Deep Neural Network

Athletes were randomly separated into training (67%, *n* = 278), validation (33%, *n* = 69), and test (33%, *n* = 70) subsets. A single matrix was created for each subset by concatenating data from all movement trials performed by all athletes in the subset. Each variable was normalized by subtracting the mean and dividing by the standard deviation of all data frames across athletes and movements in the training set for that variable. A sliding window approach was used to divide the subset data into data segments containing an equal number of data frames. The stride for the sliding window was 1/4 the window size. Each data segment was assigned a label according to the movement that was performed for the majority of the data segment. A “Null” label was included to describe times when none of the movements were being performed for a total of 14 classes.

**Figure 1:**
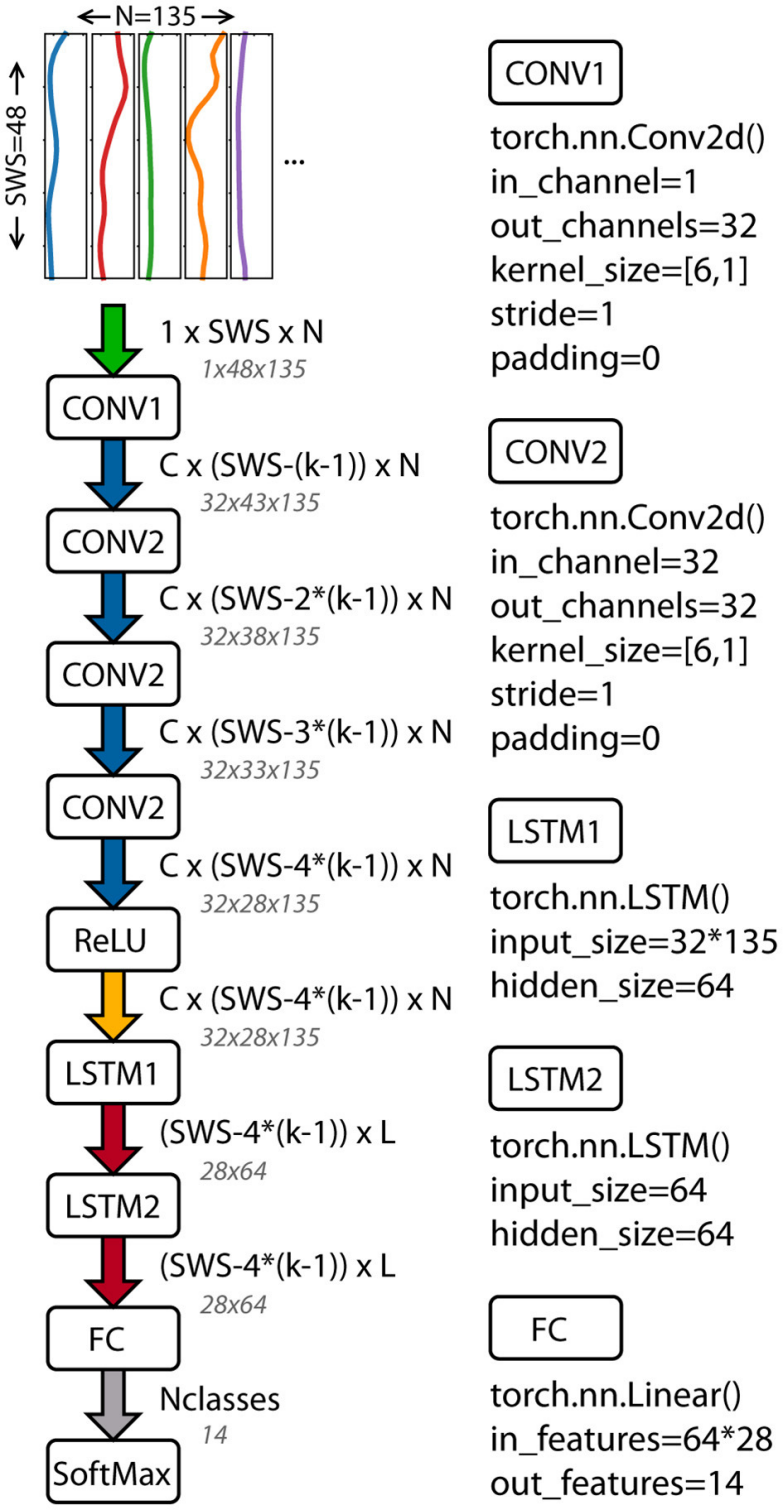
Architecture of the deep neural network used to classify athletic movements. The network combines convolutional and recurrent layers (Ordóñez and Roggen, 2016). Tensor sizes and function inputs based on the OPT data and final architecture parameters are shown. PyTorch functions and inputs are shown for each layer. SWS, sliding window size; C, number of CNN channels; N, number of columns in the input data; k, CNN kernel size; L, LSTM cells; Nclasses, number of movements classified.

A deep neural network based on the work of Ordóñez and Roggen (2016) was implemented in PyTorch (Paszke et al., 2017). The architecture combines convolutional layers to extract features with recurrent layers to model the temporal dynamics. The network includes four convolutional layers, two long-short-term memory (LSTM) recurrent layers, a linear fully connected layer, and a softmax classifier (Figure 1). The input to the network is the windowed time series data. The length of the input data was the sliding window size and the number of columns depended on the data used: 3^*^45 for OPT (x, y, and z component of each trajectory) and 5 ^*^ number of body segments for the sIMU data (3 Euler angles + 1 angular velocity norm + 1 linear acceleration norm).

For network training, a mini-batch size of 100 was used. A stochastic gradient descent (SGD) optimizer with momentum was used for training with a cross-entropy loss criterion. The DNN was trained to classify the movement performed during a given windowed data segment.

### Hyperparameter Tuning

Hyperparameter tuning was performed using a grid search with the validation set of the optical motion data (OPT). The learning parameters were tuned first as these have a larger impact on classifier performance (Hammerla et al., 2016). The learning parameters were the learning rate and momentum of the SGD optimizer. Five values of each were explored (Table 1) resulting in 25 DNNs trained on the OPT training set. The micro-averaged F1 score was calculated for the validation set to assess the performance of each DNN. The F1 score is a measure of classification accuracy that is the harmonic mean of precision and recall ($F1=2\;\cdot \frac{precision\;\cdot \;recall}{precision\;+\;recall}$). The micro-averaged F1 score calculates the mean across the classes by considering all individual predictions, which is suitable for classes of different sizes. The micro-averaged F1 score is equivalent to the micro-averaged precision, micro-averaged recall, and classification accuracy.

**Table 1:** Learning and architecture parameter values tested for hyperparameter tuning.

SGD optimizer parameter tuning					
Learning Rate	0.0001, 0.001, 0.01, 0.1, 1				
Momentum	0.5, 0.7, 0.9, 0.95, 0.98				
DNN parameter tuning					
Window Size (frames)	24				48
CNN Kernel Size (frames)	5, 6				6, 8
CNN Channels				32, 64, 96	
LSTM Cells				64, 128, 192	

After selecting the learning rate and momentum that produced the best F1 score, the architecture parameters were tuned. Two to three values were tested for each of the following parameters: sliding window size, CNN kernel filter size, CNN channels, and LSTM cells (Table 1). Note that CNN kernels of size 5 and 6 were used with window size 24 and CNN kernels of size 6 and 8 were used with window size 48. Models were assessed based on the micro-averaged F1 score.

### Comparison of Simulated IMU Sensor Data

Once the final learning and architecture parameters were determined, the final model was used to identify movements in the test set. In this case, the DNN was used individually on each athlete. All trials of athlete's data were combined and then segmented using sliding windows and the DNN was used to classify each window. Then for each frame of data, the class probabilities from each window containing that frame were averaged, and a final classification was made for that frame of data.

DNNs using the final learning and architecture parameters were also trained for the simulated IMU data on the training subset. Different combinations of simulated sensor locations were examined (Table 2). DNNs were evaluated on the test subset following the procedure outlined above. For each DNN, the confusion matrix, accuracy, precision, recall, and F1 score were calculated. Micro and macro averages and metrics for each class were produced.

**Table 2:** Combination of body segments used to train and test the DNN for the simulated IMU data.

**Data input**	**Body segments**
sIMU1	Torso
sIMU2	Torso, pelvis
sIMU3L	Torso, shanks (lower body)
sIMU3U	Torso, upper arms (upper body)
sIMU4	Torso, pelvis, thighs
sIMU4D	Forearms, shanks (distal segments)
sIMU4P	Upper arms, thighs (proximal segments)
sIMU5	Torso, forearms, shanks
sIMU13	Head, torso, pelvis, upper arms, forearms, thighs, shanks, feet

## Results

### Hyperparameter Tuning

**Figure 2:**
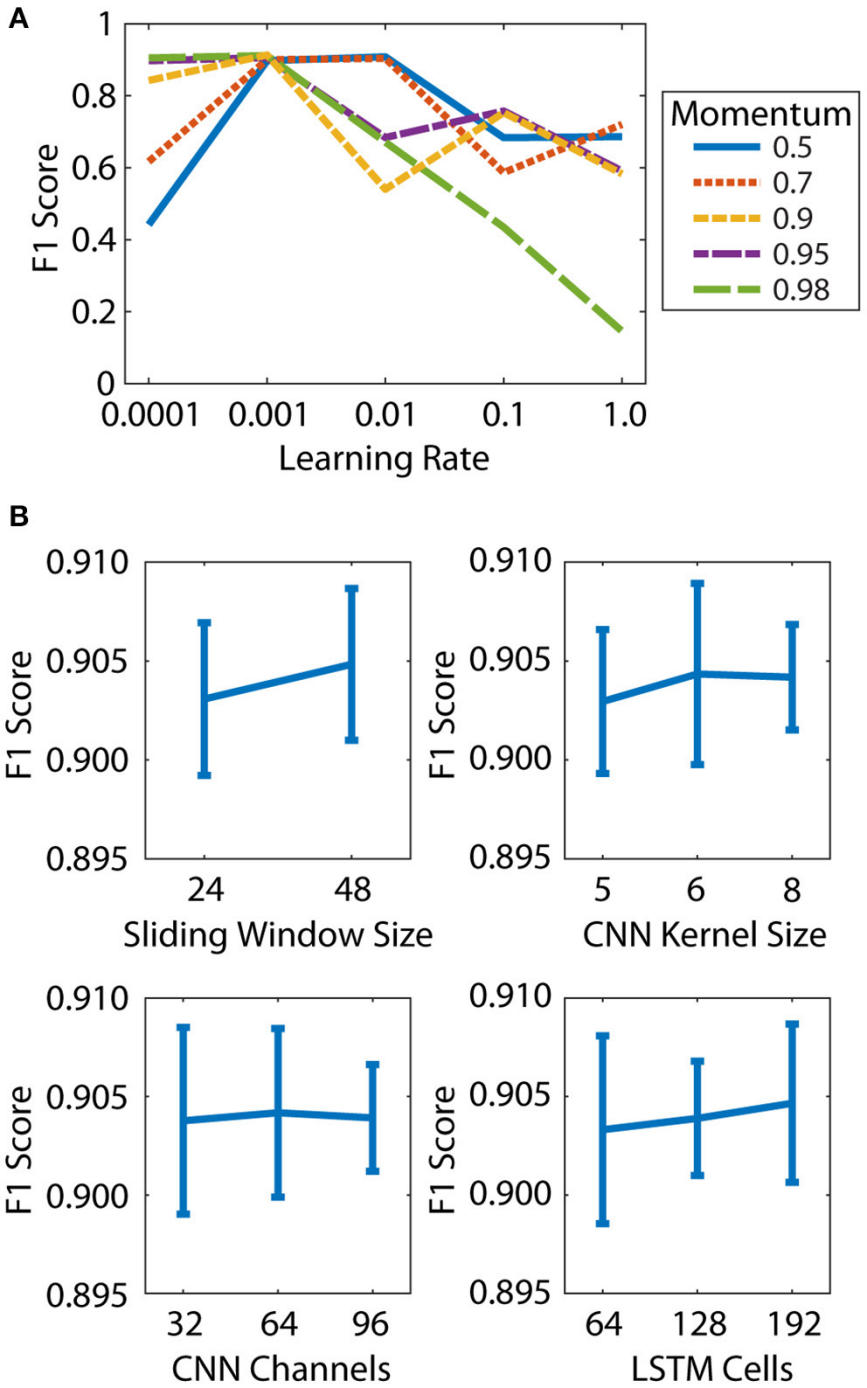
Hyperparameter tuning was performed in two steps: learning parameters **(A)** and architecture parameters **(B)**. **(A)** Effect of learning rate and momentum on micro-averaged F1 score. **(B)** Effect of sliding window size, CNN kernel size, CNN channels, and LSTM cells on micro-averaged F1 score. Mean and standard deviation of all DNNs at each parameter level are shown.

The learning rate and momentum of the SGD optimizer both had a large effect on the micro-averaged F1 score for the OPT validation set (Figure 2A). The best F1 score was obtained for a learning rate of 0.001 and momentum of 0.9, and these values were used for all subsequent models. The DNN parameters had a relatively small effect on the F1 scores, with values ranging from 0.895 to 0.911 (Figure 2B). The best results were obtained for a sliding window size of 48 (0.04 s), CNN kernel size of 6 frames, 32 CNN channels, and 64 LSTM cells. These parameters were selected for use in the final DNN.

### Comparison of Simulated IMU Sensor Data

**Figure 3:**
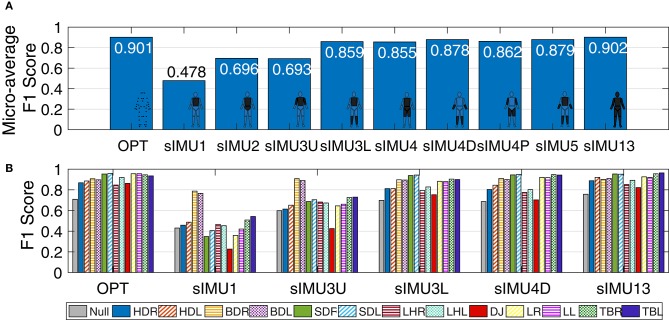
**(A)** Micro-averaged F1 score for DNNs trained using simulated IMU (sIMU) data from various combinations of body segments. **(B)** F1 score for each movement for a selection of DNNs trained on sIMU data. Scores were calculated on the test set based on classification of individual data frames. Movements are HDR/L, hop down right/left; BDR/L, bird-dog right/left; SDR/L, step-down right/left; LHR/L, L-hop right/left; DJ, drop jump; LR/L, lunge right/left; TBR/L, T-balance right/left.

Deep neural networks trained using optical data (OPT) and all 13 body segments of sIMU data (sIMU13) had similar micro-averaged F1 scores (0.901 and 0.902, respectively). In general, including more body segments improved performance (Figure 3), although only small improvements were obtained by including more than four body segments. Bird-dog (BDR/L) movements were predicted well (F1 score > 0.76) for all networks, while drop jumps (DJ) tended to be more poorly identified in general.

**Figure 4:**
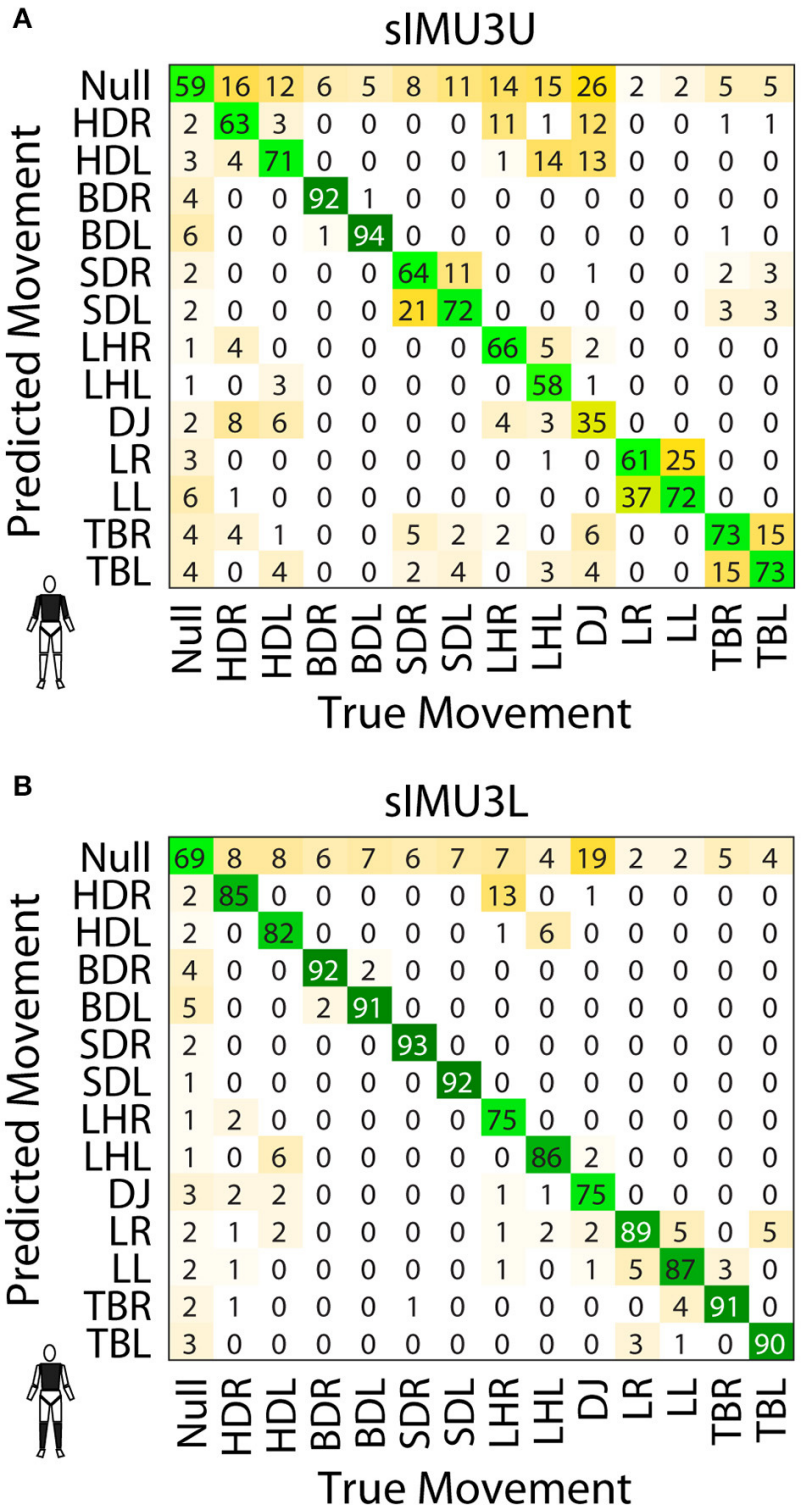
Confusion matrices for the sIMU3U **(A)** and sIMU3L **(B)** DNNs. Values are percentage of the frames of the true movement classified as the predicted movement.

The effect of including upper or lower limb data can be observed in the confusion matrices for the sIMU3U and sIMU3L models (Figure 4). With the torso and upper arms included (sIMU3U), the DNN frequently confuses left and right versions of tasks. Tasks involving jumping were also confused. The network using the torso and shanks (sIMU3L) is better able to distinguish between left and right, but occasionally confuses the T-balance (TBR/L) and lunge tasks (LR/L). L-hops (LHR/L) are sometimes classified as hop downs (HDR/L) in both three-segment networks (sIMU3L, sIMU3U).

**Figure 5:**
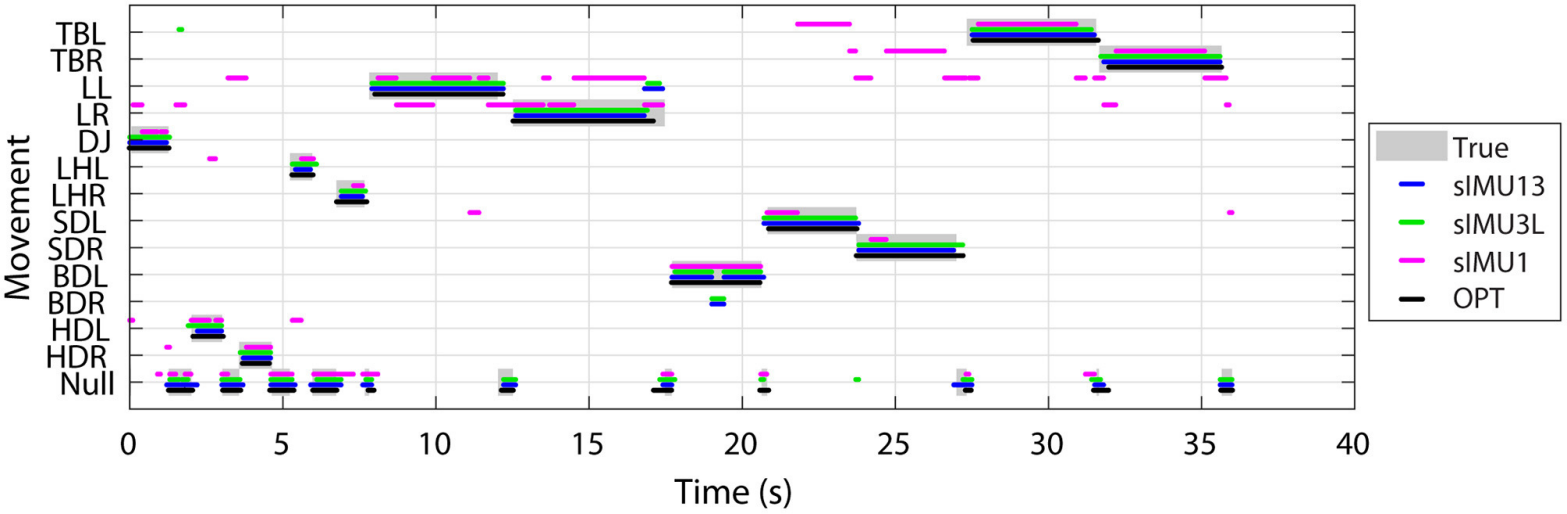
Example of movement classification for a representative athlete using DNNs trained using different sets of input data. Data collected in separate trials for each movement have been concatenated and displayed continuously.

The true and predicted movements over time for the OPT, sIMU1, sIMU3L, and sIMU13 models are shown in Figure 5 for a representative athlete. OPT, sIMU13, and sIMU3L were better able to predict the entire duration of movements. Networks with fewer body segments tended to switch between predictions. The misclassification between movements and Null largely occurs at the beginning and end of a movement.

The complete set of precision, recall, F1 scores, and confusion matrices are included in the Supplementary Material.

## Discussion

The deep neural network (DNN) combining convolutional and recurrent layers was able to successfully identify athletic movements for both optical motion capture trajectories and simulated inertial measurement unit (sIMU) data. DNNs trained using optical motion capture data (OPT) and full body simulated IMU (sIMU13) data had similar performance with F1 scores of approximately 0.90. Classification accuracy was poor (<70%) if fewer than three body segments were included or the lower limbs were not included in the sIMU data.

There was minimal difference between micro-averaged F1 scores for the DNNs trained using five or 13 body segments. This indicates that it is not necessary to include measurements from the head or more than one segment from each upper or lower limb. This is encouraging as the use of fewer sensors would simplify the set-up before a movement screen and would be less of a hindrance to the athlete's motion. The F1 score for sIMU3L, which used the torso and shanks, was only ~0.04 less than for the full body DNN. Therefore, depending on the desired accuracy, classification rates may be sufficient using only three sensors.

Some movements, such as the bird-dogs, were more easily identified by the DNN, even for networks trained on sIMU data from one or two segments. This is likely because trunk motion for these movements is substantially different from that of the other movements, with the trunk horizontal and relatively stationary throughout the motion. Including arm segments prevented confusion between T-balances and lunges. The drop jump was classified particularly poorly when few segments were used, often being classified as the null condition. This may in part be due to the way the start and end of the L-hop motion was defined. The L-hop involved the athlete jumping horizontally forward, landing on one foot, then jumping laterally and landing on the opposite foot. This movement was defined to begin when the athlete had reached their maximum height during the initial jump. Without sufficient data, the DNN was unable to differentiate between the end of the drop jump movement, which involved a vertical jump, and the initial jump of the L-hop which was included in the null condition.

The F1 score we achieved in classifying athletic movements is similar to previously reported human activity classification results. The architecture of the DNN used in this study was based on the work of Ordóñez and Roggen (2016), who achieved an F1 score of 0.895 on a dataset including various modes of locomotion. Other work has reported classification accuracies ranging from 83 to 100% for everyday activities (Pärkkä et al., 2006; Yeoh et al., 2008; Attal et al., 2015; Yang et al., 2015) and 79–93% for movements involved in various sports (Schuldhaus et al., 2015; Groh et al., 2016; Anand et al., 2017; Cust et al., 2019).

Previous work on classification of everyday activities, such as walking, jogging, sitting, stair climbing, etc., has identified one sensor placed at the waist as producing the best classification accuracy (Cleland et al., 2013; Pannurat et al., 2017). In the current study, we found that a single simulated torso sensor resulted in a poor classification accuracy of 48%. This discrepancy can likely be attributed to the differences in activities included, as optimal sensor placement depends on the activity (Atallah et al., 2011; Attal et al., 2015). The activities classified in the previous studies involve activities that are repetitive and take place over a relatively long period of time. The athletic movements included in our study, however, are short single movements. Furthermore, the need to differentiate right and left versions of the movements makes classification with a single torso-mounted sensor more challenging.

The sIMU DNNs relied on simulated IMU data generated based on optical motion tracking markers. Therefore, these results likely represent a best-case scenario for classification of these athletic movements using wearable sensors. Sensor drift is a common issue with IMUs and therefore it is possible that misclassification rates would be larger using real sensor data, particularly for long data collections as drift increases over time. Care would also need to be taken to standardize sensor placement on each body segment. While we have used the Euclidean norm of the angular velocity and linear acceleration, error would be introduced into the angular orientation of the body segments by misaligned sensors. Additionally, it may be possible to mitigate sensor misalignment issues using a static or dynamic calibration at the beginning of the data collection. Despite the reliance on simulated sensor data, the results presented here highlight the potential for movement classification using wearable sensors and provide guidance for sensor placement in future work.

In this study, separate data trials were recorded for each motion and these were combined for the classification. As a result, the amount of null data frames included was relatively small. It may be necessary to included more null condition training data, including transitions between movements, for the DNN to be used successfully on continuously collected data.

Accurate classification of movements is critical for this DNN approach to be used with no manual intervention in combination with data-driven assessments of movement quality, as the quality could only be assessed on properly identified movements. Some errors may be possible to correct with additional processing, such as when the classification jumps to another movement for a few frames in the middle of an otherwise accurately classified movement. We observed that a large source of error was over- or under-estimating the start or end points of a movement with misclassification between the movement and the null condition. It is possible that movement quality could still be quantified with these slight errors in start and end points, but future work will be required to verify this. Alternately, a small amount of manual intervention could be used to verify task identification before proceeding to quantification of movement quality.

The favorable classification rates obtained in this work using simulated sensor data demonstrates the feasibility of classifying athletic tasks typical of movement screens using wearable sensors. Using simulated IMU data, we observed the best classification accuracy by including data from all body segments; however, we obtained good results using as few as three simulated sensors. This indicates that classification of these athletic movements using real IMU data would require at least three sensors and should include the torso and legs. Implementation of a movement classification DNN with wearable sensor data would facilitate automatic data-driven assessment of movement quality, eliminating subjective scoring, and increasing the ability to detect subtle differences.

## Data Availability Statement

Code, sample data, and trained DNNs weights are available at doi: 10.5281/zenodo.3546204.

## Ethics Statement

The studies involving human participants were reviewed and approved by University of Ottawa Research Ethics Board. Written informed consent to participate in this study was provided by the participant or their legal guardian/next of kin.

## Author Contributions

AC, GR, and RG conceived of the study, interpreted the results, and critically revised the manuscript. GR collected and processed the data. AC implemented the neural network, analyzed the results, and prepared the manuscript.

### Conflict of Interest

The authors declare that the research was conducted in the absence of any commercial or financial relationships that could be construed as a potential conflict of interest.
