# Supplementary material for: Sensor Data Required for Automatic Recognition of Athletic Tasks Using Deep Neural Networks
Source: Front Bioeng Biotechnol. 2020 Jan 21;7:473. doi: 10.3389/fbioe.2019.00473 (PMC6985033; doi:10.3389/fbioe.2019.00473)
Supplement: Supplementary file 1 [file Data_Sheet_1.DOCX]

Supplementary Material

# Final DNN classification performance metrics

Precision, recall, F1 scores, and the receiver operating characteristic area under the curve (ROC AUC) were calculated for each movement with each of the DNNs trained on optical or simulated IMU data (Tables S1-S10). The micro- and macro-averaged values for each of these was also calculated. Confusion matrices for each matrix are presented in Figures S1-S10.

## OPT

Table S1: Classification performance metrics for marker trajectories (OPT)

| **Movement** | **Precision** | **Recall** | **F1 Score** | **ROC AUC** | **Accuracy** |
| --- | --- | --- | --- | --- | --- |
| Null | 0.763 | 0.661 | 0.709 | 0.808 | 0.901 |
| HDR | 0.822 | 0.919 | 0.868 | 0.981 |  |
| HDL | 0.846 | 0.929 | 0.886 | 0.988 |  |
| RSR | 0.881 | 0.935 | 0.907 | 0.996 |  |
| RSL | 0.881 | 0.913 | 0.897 | 0.996 |  |
| SDR | 0.935 | 0.971 | 0.953 | 0.999 |  |
| SDL | 0.955 | 0.962 | 0.958 | 0.998 |  |
| LHR | 0.900 | 0.799 | 0.846 | 0.981 |  |
| LHL | 0.926 | 0.915 | 0.920 | 0.989 |  |
| DJ | 0.803 | 0.932 | 0.863 | 0.996 |  |
| LR | 0.955 | 0.956 | 0.956 | 0.995 |  |
| LL | 0.956 | 0.955 | 0.956 | 0.995 |  |
| TBR | 0.946 | 0.945 | 0.945 | 0.998 |  |
| TBL | 0.919 | 0.951 | 0.935 | 0.997 |  |
| **Micro** | 0.901 | 0.901 | 0.901 | 0.990 |  |
| **Macro** | 0.892 | 0.910 | 0.900 | 0.980 |  |

Figure S1: Confusion matrix for the OPT DNN. Percentage is the percent of true movement frames predicted to be the predicted movement. Total data frames classified as the predicted movement is also given.

## sIMU1

## Table S2: Classification performance metrics simulated IMU data for the torso (sIMU1)

| **Movement** | **Precision** | **Recall** | **F1 Score** | **ROC AUC** | **Accuracy** |
| --- | --- | --- | --- | --- | --- |
| Null | 0.520 | 0.367 | 0.431 | 0.714 | 0.478 |
| HDR | 0.488 | 0.432 | 0.458 | 0.949 |  |
| HDL | 0.473 | 0.502 | 0.487 | 0.960 |  |
| RSR | 0.737 | 0.845 | 0.788 | 0.986 |  |
| RSL | 0.696 | 0.852 | 0.766 | 0.980 |  |
| SDR | 0.430 | 0.292 | 0.348 | 0.857 |  |
| SDL | 0.434 | 0.380 | 0.405 | 0.880 |  |
| LHR | 0.499 | 0.436 | 0.465 | 0.963 |  |
| LHL | 0.566 | 0.378 | 0.453 | 0.962 |  |
| DJ | 0.493 | 0.147 | 0.227 | 0.906 |  |
| LR | 0.360 | 0.359 | 0.360 | 0.781 |  |
| LL | 0.329 | 0.583 | 0.421 | 0.805 |  |
| TBR | 0.645 | 0.419 | 0.508 | 0.864 |  |
| TBL | 0.517 | 0.570 | 0.542 | 0.848 |  |
| **Micro** | 0.478 | 0.478 | 0.478 | 0.902 |  |
| **Macro** | 0.513 | 0.469 | 0.476 | 0.890 |  |

Figure S2: Confusion matrix for the sIMU1 DNN. Percentage is the percent of true movement frames predicted to be the predicted movement. Total data frames classified as the predicted movement is also given.

## sIMU2

Table S3: Classification performance metrics simulated IMU data for the torso and pelvis (sIMU2)

| **Movement** | **Precision** | **Recall** | **F1 Score** | **ROC AUC** | **Accuracy** |
| --- | --- | --- | --- | --- | --- |
| Null | 0.601 | 0.524 | 0.560 | 0.788 | 0.696 |
| HDR | 0.651 | 0.646 | 0.648 | 0.972 |  |
| HDL | 0.659 | 0.652 | 0.656 | 0.979 |  |
| RSR | 0.874 | 0.872 | 0.873 | 0.990 |  |
| RSL | 0.823 | 0.877 | 0.849 | 0.983 |  |
| SDR | 0.670 | 0.628 | 0.649 | 0.941 |  |
| SDL | 0.655 | 0.656 | 0.655 | 0.942 |  |
| LHR | 0.706 | 0.489 | 0.578 | 0.972 |  |
| LHL | 0.689 | 0.575 | 0.627 | 0.975 |  |
| DJ | 0.646 | 0.503 | 0.566 | 0.955 |  |
| LR | 0.607 | 0.660 | 0.632 | 0.894 |  |
| LL | 0.606 | 0.660 | 0.632 | 0.902 |  |
| TBR | 0.835 | 0.881 | 0.857 | 0.982 |  |
| TBL | 0.806 | 0.831 | 0.819 | 0.969 |  |
| **Micro** | 0.696 | 0.696 | 0.696 | 0.954 |  |
| **Macro** | 0.702 | 0.675 | 0.686 | 0.946 |  |

Figure S3: Confusion matrix for the sIMU2 DNN. Percentage is the percent of true movement frames predicted to be the predicted movement. Total data frames classified as the predicted movement is also given.

## sIMU3L

Table S4: Classification performance metrics simulated IMU data for the torso and shanks (sIMU3L)

| **Movement** | **Precision** | **Recall** | **F1 Score** | **ROC AUC** | **Accuracy** |
| --- | --- | --- | --- | --- | --- |
| Null | 0.703 | 0.694 | 0.698 | 0.856 | 0.859 |
| HDR | 0.773 | 0.854 | 0.811 | 0.984 |  |
| HDL | 0.807 | 0.816 | 0.811 | 0.987 |  |
| RSR | 0.877 | 0.918 | 0.897 | 0.995 |  |
| RSL | 0.877 | 0.914 | 0.895 | 0.992 |  |
| SDR | 0.945 | 0.931 | 0.938 | 0.997 |  |
| SDL | 0.963 | 0.924 | 0.943 | 0.996 |  |
| LHR | 0.844 | 0.750 | 0.794 | 0.995 |  |
| LHL | 0.796 | 0.863 | 0.828 | 0.993 |  |
| DJ | 0.758 | 0.748 | 0.753 | 0.982 |  |
| LR | 0.880 | 0.885 | 0.883 | 0.968 |  |
| LL | 0.891 | 0.868 | 0.880 | 0.960 |  |
| TBR | 0.899 | 0.909 | 0.904 | 0.987 |  |
| TBL | 0.902 | 0.897 | 0.899 | 0.989 |  |
| **Micro** | 0.859 | 0.859 | 0.859 | 0.982 |  |
| **Macro** | 0.851 | 0.855 | 0.852 | 0.977 |  |

Figure S4: Confusion matrix for the sIMU3L DNN. Percentage is the percent of true movement frames predicted to be the predicted movement. Total data frames classified as the predicted movement is also given.

## sIMU3U

Table S5: Classification performance metrics simulated IMU data for the torso and upper arms (sIMU3U)

| **Movement** | **Precision** | **Recall** | **F1 Score** | **ROC AUC** | **Accuracy** |
| --- | --- | --- | --- | --- | --- |
| Null | 0.612 | 0.585 | 0.598 | 0.708 | 0.693 |
| HDR | 0.601 | 0.627 | 0.614 | 0.969 |  |
| HDL | 0.599 | 0.714 | 0.651 | 0.978 |  |
| RSR | 0.893 | 0.923 | 0.908 | 0.996 |  |
| RSL | 0.847 | 0.940 | 0.891 | 0.994 |  |
| SDR | 0.735 | 0.644 | 0.687 | 0.969 |  |
| SDL | 0.686 | 0.723 | 0.704 | 0.967 |  |
| LHR | 0.702 | 0.663 | 0.682 | 0.981 |  |
| LHL | 0.807 | 0.577 | 0.673 | 0.981 |  |
| DJ | 0.542 | 0.349 | 0.425 | 0.929 |  |
| LR | 0.681 | 0.613 | 0.645 | 0.935 |  |
| LL | 0.611 | 0.720 | 0.661 | 0.931 |  |
| TBR | 0.720 | 0.732 | 0.726 | 0.939 |  |
| TBL | 0.731 | 0.728 | 0.730 | 0.939 |  |
| **Micro** | 0.693 | 0.693 | 0.693 | 0.957 |  |
| **Macro** | 0.698 | 0.681 | 0.685 | 0.944 |  |

Figure S5: Confusion matrix for the sIMU3U DNN. Percentage is the percent of true movement frames predicted to be the predicted movement. Total data frames classified as the predicted movement is also given.

## sIMU4

Table S6: Classification performance metrics simulated IMU data for the torso, pelvis, and thighs (sIMU4)

| **Movement** | **Precision** | **Recall** | **F1 Score** | **ROC AUC** | **Accuracy** |
| --- | --- | --- | --- | --- | --- |
| Null | 0.725 | 0.680 | 0.702 | 0.844 | 0.693 |
| HDR | 0.817 | 0.901 | 0.857 | 0.992 |  |
| HDL | 0.792 | 0.910 | 0.847 | 0.994 |  |
| RSR | 0.903 | 0.919 | 0.911 | 0.993 |  |
| RSL | 0.898 | 0.921 | 0.909 | 0.993 |  |
| SDR | 0.931 | 0.928 | 0.929 | 0.994 |  |
| SDL | 0.919 | 0.942 | 0.930 | 0.996 |  |
| LHR | 0.839 | 0.799 | 0.819 | 0.992 |  |
| LHL | 0.870 | 0.812 | 0.840 | 0.990 |  |
| DJ | 0.787 | 0.787 | 0.787 | 0.983 |  |
| LR | 0.854 | 0.811 | 0.832 | 0.950 |  |
| LL | 0.815 | 0.855 | 0.834 | 0.958 |  |
| TBR | 0.907 | 0.937 | 0.922 | 0.993 |  |
| TBL | 0.936 | 0.914 | 0.925 | 0.991 |  |
| **Micro** | 0.855 | 0.855 | 0.855 | 0.982 |  |
| **Macro** | 0.857 | 0.865 | 0.860 | 0.976 |  |

Figure S6: Confusion matrix for the sIMU4 DNN. Percentage is the percent of true movement frames predicted to be the predicted movement. Total data frames classified as the predicted movement is also given.

## sIMU4D

Table S7: Classification performance metrics simulated IMU data for the forearms and shanks (sIMU4D)

| **Movement** | **Precision** | **Recall** | **F1 Score** | **ROC AUC** | **Accuracy** |
| --- | --- | --- | --- | --- | --- |
| Null | 0.723 | 0.654 | 0.687 | 0.847 | 0.878 |
| HDR | 0.807 | 0.800 | 0.804 | 0.984 |  |
| HDL | 0.812 | 0.880 | 0.845 | 0.989 |  |
| RSR | 0.888 | 0.933 | 0.910 | 0.995 |  |
| RSL | 0.876 | 0.925 | 0.900 | 0.996 |  |
| SDR | 0.944 | 0.945 | 0.944 | 0.997 |  |
| SDL | 0.946 | 0.951 | 0.948 | 0.998 |  |
| LHR | 0.826 | 0.729 | 0.775 | 0.993 |  |
| LHL | 0.851 | 0.758 | 0.802 | 0.992 |  |
| DJ | 0.665 | 0.745 | 0.703 | 0.975 |  |
| LR | 0.928 | 0.914 | 0.921 | 0.987 |  |
| LL | 0.907 | 0.929 | 0.918 | 0.984 |  |
| TBR | 0.933 | 0.962 | 0.947 | 0.997 |  |
| TBL | 0.937 | 0.949 | 0.943 | 0.996 |  |
| **Micro** | 0.878 | 0.878 | 0.878 | 0.987 |  |
| **Macro** | 0.860 | 0.862 | 0.860 | 0.981 |  |

Figure S7: Confusion matrix for the sIMU4D DNN. Percentage is the percent of true movement frames predicted to be the predicted movement. Total data frames classified as the predicted movement is also given.

## sIMU4P

Table S8: Classification performance metrics simulated IMU data for the upper arms and thighs (sIMU4P)

| **Movement** | **Precision** | **Recall** | **F1 Score** | **ROC AUC** | **Accuracy** |
| --- | --- | --- | --- | --- | --- |
| Null | 0.691 | 0.738 | 0.714 | 0.862 | 0.862 |
| HDR | 0.799 | 0.880 | 0.838 | 0.993 |  |
| HDL | 0.858 | 0.840 | 0.849 | 0.994 |  |
| RSR | 0.880 | 0.926 | 0.902 | 0.993 |  |
| RSL | 0.913 | 0.862 | 0.887 | 0.993 |  |
| SDR | 0.953 | 0.952 | 0.952 | 0.997 |  |
| SDL | 0.956 | 0.935 | 0.945 | 0.998 |  |
| LHR | 0.816 | 0.737 | 0.775 | 0.986 |  |
| LHL | 0.821 | 0.757 | 0.788 | 0.989 |  |
| DJ | 0.745 | 0.642 | 0.690 | 0.975 |  |
| LR | 0.870 | 0.859 | 0.864 | 0.973 |  |
| LL | 0.851 | 0.866 | 0.859 | 0.963 |  |
| TBR | 0.918 | 0.954 | 0.936 | 0.994 |  |
| TBL | 0.971 | 0.895 | 0.931 | 0.995 |  |
| **Micro** | 0.862 | 0.862 | 0.862 | 0.983 |  |
| **Macro** | 0.860 | 0.846 | 0.852 | 0.979 |  |

Figure S8: Confusion matrix for the sIMU4P DNN. Percentage is the percent of true movement frames predicted to be the predicted movement. Total data frames classified as the predicted movement is also given.

## sIMU5

Table S9: Classification performance metrics simulated IMU data for the torso, forearms, and shanks (sIMU5)

| **Movement** | **Precision** | **Recall** | **F1 Score** | **ROC AUC** | **Accuracy** |
| --- | --- | --- | --- | --- | --- |
| Null | 0.722 | 0.665 | 0.692 | 0.858 | 0.879 |
| HDR | 0.799 | 0.809 | 0.804 | 0.987 |  |
| HDL | 0.800 | 0.859 | 0.829 | 0.988 |  |
| RSR | 0.914 | 0.895 | 0.904 | 0.995 |  |
| RSL | 0.895 | 0.928 | 0.911 | 0.993 |  |
| SDR | 0.949 | 0.939 | 0.944 | 0.998 |  |
| SDL | 0.965 | 0.910 | 0.937 | 0.996 |  |
| LHR | 0.818 | 0.791 | 0.804 | 0.994 |  |
| LHL | 0.866 | 0.801 | 0.832 | 0.994 |  |
| DJ | 0.739 | 0.785 | 0.761 | 0.982 |  |
| LR | 0.912 | 0.919 | 0.915 | 0.989 |  |
| LL | 0.904 | 0.913 | 0.909 | 0.983 |  |
| TBR | 0.936 | 0.963 | 0.950 | 0.996 |  |
| TBL | 0.908 | 0.972 | 0.939 | 0.997 |  |
| **Micro** | 0.879 | 0.879 | 0.879 | 0.987 |  |
| **Macro** | 0.866 | 0.868 | 0.866 | 0.982 |  |

Figure S9: Confusion matrix for the sIMU1 DNN. Percentage is the percent of true movement frames predicted to be the predicted movement. Total data frames classified as the predicted movement is also given.

## sIMU13

Table S10: Classification performance metrics simulated IMU data for all body segments: head, torso, pelvis, upper arms, forearms, thighs, shanks, and feet (sIMU13)

| **Movement** | **Precision** | **Recall** | **F1 Score** | **ROC AUC** | **Accuracy** |
| --- | --- | --- | --- | --- | --- |
| Null | 0.741 | 0.775 | 0.757 | 0.893 | 0.902 |
| HDR | 0.873 | 0.903 | 0.888 | 0.996 |  |
| HDL | 0.930 | 0.911 | 0.921 | 0.996 |  |
| RSR | 0.879 | 0.923 | 0.901 | 0.995 |  |
| RSL | 0.892 | 0.925 | 0.908 | 0.994 |  |
| SDR | 0.964 | 0.942 | 0.953 | 0.999 |  |
| SDL | 0.961 | 0.941 | 0.951 | 0.998 |  |
| LHR | 0.867 | 0.836 | 0.851 | 0.993 |  |
| LHL | 0.944 | 0.847 | 0.893 | 0.993 |  |
| DJ | 0.849 | 0.796 | 0.821 | 0.982 |  |
| LR | 0.921 | 0.930 | 0.926 | 0.990 |  |
| LL | 0.933 | 0.908 | 0.920 | 0.990 |  |
| TBR | 0.958 | 0.952 | 0.955 | 0.997 |  |
| TBL | 0.977 | 0.951 | 0.964 | 0.998 |  |
| **Micro** | 0.902 | 0.902 | 0.902 | 0.992 |  |
| **Macro** | 0.906 | 0.896 | 0.901 | 0.987 |  |

Figure S10: Confusion matrix for the sIMU1 DNN. Percentage is the percent of true movement frames predicted to be the predicted movement. Total data frames classified as the predicted movement is also given.
